# Gene expression profiling by microarrays – clinical implications

**DOI:** 10.1038/sj.bjc.6603486

**Published:** 2007-01-23

**Authors:** J-P Gillet

**Affiliations:** 1 Center for Cancer Research, NCI, NIH, Bethesda, MD, USA

The development of the DNA microarray, a novel tool assessing the simultaneous expression profile of hundreds to thousands of genes, has revolutionised molecular biology. Since 1995 and the first report of the elaboration of a high-throughput system monitoring the expression of many genes in only one experiment by Patrick O Brown, the published papers based on this technology have proliferated.

In the past 6 years or so, microarray technology has been extensively used to investigate numerous human tumour types and other pathologies. Numerous publications reporting microarray-generated results have significantly contributed to a better understanding of the molecular events occurring in various pathologies and have provided important insights into the true pathobiologic nature of the diseases.

The present survey entitled ‘Gene expression profiling by microarray – clinical implications’ provides nine chapters which have been written by 26 contributors under the coordination of Professor Wolf-Karsten Hofmann, who has been instrumental in unraveling the pathobiology of leukaemia and lymphoma using microarray technology. The stated purpose of this book is to serve as a guide to help scientists better understand the overwhelming number of publications reporting microarray-based studies. It is well written, appropriately illustrated, easy to read and some chapters are highly interesting, depending on the expertise and focus of the reader. Each chapter provides an extensive overview of the specific topic discussed and up-to-date references are used. The first three chapters are particularly interesting, as they provide a thorough review of the technology, concluded by a critical analysis of the current advantages and drawbacks linked to microarray analysis. In these sections, the authors evaluate the different microarray platforms, probe labeling, image analysis, various crucial and essential quality controls, etc.

Owing to the large amount of data generated by microarray experiments, statistical analysis should be considered as the cornerstone of such studies. Therefore, chapter 3, entitled, ‘Statistical analysis of gene expression data’ is the most important one and should be dissected in each and every detail by an investigator running this kind of assay. The review is clear and accessible to a nonspecialised biostatistician.

Current diagnostics are not sufficient to accurately identify discrete differences in tumours sharing the same origin or sharing identical histopathological markers. As a result, treatments of similar cancers diagnosed by conventional histopathological criteria can have radically different outcomes. For this reason, gene expression profiling can be exploited to improve not only the accuracy of a diagnosis, but can also help to select the appropriate treatment, which ultimately can lead to a more favourable prognosis. The last six chapters cover the different kinds of applications suggested above and made possible by microarray technology. Indeed, the authors review some recent insights into five diseases (heart failure and diabetes, together with breast cancer, leukaemia and lymphoma) achieved by microarray gene expression profiling. The last section provides a survey of the recent studies focused on gene expression signatures and their significance regarding the clinical outcome and the response to specific drug regimens. This chapter discusses the outstanding results obtained in non-Hodgkin's lymphoma, acute lymphoblastic leukaemia, breast cancer and in some other solid tumours, these latter being briefly overviewed.

Although I have no major concerns about this book, one minor problem is the lack of a final discussion that provides critical evaluation and summarises the views of the panel of experts. This leaves the reader a bit unsatisfied. Nevertheless, interesting future perspectives are delineated throughout the different sections. Haferlach and co-workers mentioned the beginning of a further trial conducted by the European Leukaemia Network using 10 sites and 4000 arrays to validate the microarray approach for diagnosis, prognosis and treatment decisions. Although these kinds of international trials can lead to the use of a standard microarray platform, the introduction of the technology in routine clinical application is still debated. Detection of gene expression alone does not necessarily indicate that a protein is present. To provide complete information, an interesting approach termed system biology, encompassing analysis of an individual's complete biological system, has been suggested. Individualised therapy is expected to emerge from this kind of study.

In conclusion, this is a well-written and informative book that covers all the important aspects of the field. It is recommended to readers who desire an overview of the field and to those who want an overview of a specialised topic as well. I believe the authors have reached their goal; this work will definitely help readers to make sense of the large number of publications reporting DNA microarray data.

